# Survey of Current Management Practices for Carotid Webs

**DOI:** 10.31486/toj.18.0114

**Published:** 2019

**Authors:** Kyle Wojcik, James Milburn, Gabriel Vidal, Joseph Tarsia, Andrew Steven

**Affiliations:** ^1^ The University of Queensland Faculty of Medicine, Ochsner Clinical School, New Orleans, LA; ^2^ Department of Radiology, Ochsner Clinic Foundation, New Orleans, LA; ^3^ Department of Neurology, Ochsner Clinic Foundation, New Orleans, LA

**Keywords:** *Angiography–digital subtraction*, *carotid artery diseases*, *carotid artery–internal*, *cerebral angiography*, *diagnostic imaging*, *magnetic resonance spectroscopy*, *stroke*, *tomography–x-ray computed*

## Abstract

**Background:** Carotid webs are thick, fibrous intimal bands that appear as intraluminal shelf-like defects at the carotid bifurcation on vascular imaging. These lesions are a potential underrecognized cause of cryptogenic ischemic stroke. Although the recognition of carotid webs has increased, no evidence-based treatment guidelines are available. We surveyed subspecialists across multiple neurologic disciplines to assess the state of current clinical practice.

**Methods:** An 8-question multiple-choice style survey of neurologists and radiologists assessed familiarity with this disease entity, preferred imaging modalities, and management strategies for asymptomatic and symptomatic (producing stroke) carotid webs. Responses were collected through SurveyMonkey software via anonymous responses to a posted survey link on the Society of Neurointerventional Surgery website in addition to invitation emails sent to colleagues in corresponding fields.

**Results:** Of the 74 total respondents, 64% identified as neurointerventionalists. Respondents identified computed tomography angiography as the most commonly used imaging modality to place carotid webs in the differential diagnosis (57% of respondents’ preference), while conventional digital subtraction angiogram was the preferred modality to confirm a web (54% of respondents’ preference). Respondents preferred single and dual antiplatelet therapy to manage asymptomatic and acute stroke-producing carotid webs, while invasive treatment was most commonly sought for webs producing recurrent strokes.

**Conclusion:** Familiarity with carotid webs varied across subspecialties. We found some consensus among respondents on the imaging modality preferred to identify webs, on asymptomatic carotid web management, and on recurrently symptomatic (multiple strokes) carotid web management. Less consistency was seen regarding preferences for confirmatory imaging and management of acutely symptomatic (initial stroke) carotid webs.

## INTRODUCTION

Momose and New first used the word *web* in 1973 to describe an intravascular finding on angiography.^1^ Four years later, Osborn and Anderson identified a “smooth, well-defined web” on angiography while examining patients with fibromuscular dysplasia (FMD).^2^ Since that time, carotid webs have been described as nonatherosclerotic fibrous bands that arise along the posterior margin of the carotid bulb.^3-7^

Histopathologically, these entities are characterized by fibroelastic thickening of the arterial intima^6^ and appear on imaging modalities as shelf-like or triangular-shaped intraluminal projections. Carotid webs also have been referred to as an atypical variant of FMD, with intimal fibrosis and hyperplasia on histology in contrast to the classic, medial variant.^8-10^ Typically, FMD occurs in middle-aged women with a classic “string of beads” imaging appearance and does not have a direct association with ischemic stroke.^9,10^

The prevalence of these lesions is estimated to be approximately 1%, although they may be overlooked in clinical practice as they are not typically associated with significant vascular stenosis. The significance of these lesions is not yet known, but evidence is emerging of an association with ischemic strokes.^3,11-14^ Up to one-third of all patients presenting with ischemic strokes lack an identifiable cause and are classified as cryptogenic in etiology, with most of these cases occurring in young patients without traditional vascular risk factors. Carotid webs are not typically associated with flow-limiting stenosis; however, they are thought to result in turbulent flow with the projection serving as a nidus for thrombus formation.^6^

Despite the increasing recognition of carotid webs, evidence-based management guidelines are limited. Only a few studies have investigated management outcomes,^12-19^ with most of these assessing a single treatment option.

We developed a survey to address a gap in the literature: identifying which specialists and subspecialists are encountering carotid webs, their familiarity with this rare disease entity, and their management preferences for carotid webs in multiple scenarios.

## METHODS

The goal of our survey was to assess the familiarity with and management of carotid webs among physicians who are most likely to have an impact on patients with carotid webs. The authors, representing multiple subspecialties, developed the 8 multiple-choice questions included on the survey. A survey link was posted at the Society of Neurointerventional Surgery website (available online for 1 month at www.snisonline.org), and emails requesting participation were sent to colleagues of the authors. All responses were collected anonymously through SurveyMonkey software.

## RESULTS

Of the 74 total respondents, the majority (64%) identified as neurointerventionalists. The Table presents the responses to the survey questions by subspecialty and overall.

**Table.** Carotid Web Survey Responses by Subspecialty and Overall

	Type of Respondent				
Survey Question/Response	General Neurologist	Vascular Neurologist	Neuroradiologist	Neurointerventionalist	All
1. In what capacity do you practice? (select all that apply)					
General neurologist					6 (8)
Vascular neurologist					11 (15)
Neuroradiologist					10 (14)
Neurointerventionalist					47 (64)
2. How familiar are you with this disease entity?					
Never heard of it	1 (17)	1 (9)	0 (0)	3 (7)	5 (7)
Limited clinical practice	5 (83)	4 (36)	6 (60)	17 (38)	32 (44)
Routine clinical practice	0 (0)	5 (45)	4 (40)	24 (53)	33 (46)
Extensive clinical practice	0 (0)	1 (9)	0 (0)	1 (2)	2 (3)
3. How frequently do you encounter carotid webs?					
Never	5 (83)	3 (27)	1 (10)	7 (15)	16 (22)
1-2 cases per year	1 (17)	2 (18)	5 (50)	21 (45)	29 (39)
3-5 cases per year	0 (0)	2 (18)	2 (20)	13 (28)	17 (23)
6-10 cases per year	0 (0)	4 (36)	2 (20)	3 (6)	9 (12)
>10 cases per year	0 (0)	0 (0)	0 (0)	3 (6)	3 (4)
4. What imaging modality most commonly put carotid web in the differential diagnosis (even if further imaging was sought)?					
(select all that apply)					
Conventional DSA	0 (0)	4 (31)	2 (13)	15 (23)	21 (21)
CTA	3 (43)	8 (62)	10 (63)	37 (57)	58 (57)
MRA contrast enhanced	1 (14)	0 (0)	2 (13)	7 (11)	10 (10)
MRA time of flight	0 (0)	0 (0)	1 (6)	2 (3)	3 (3)
MRI vessel wall imaging	0 (0)	0 (0)	1 (6)	1 (2)	2 (2)
Ultrasound Doppler	0 (0)	0 (0)	0 (0)	3 (5)	3 (3)
Other (please specify)^a^	3 (43)	1 (8)	0 (0)	0 (0)	4 (4)
5. Which imaging modalities do you prefer to confirm the diagnosis of carotid web? (select all that apply)					
Conventional DSA	1 (13)	9 (69)	4 (36)	36 (59)	50 (54)
CTA	2 (25)	2 (15)	6 (55)	13 (21)	23 (25)
MRA contrast enhanced	2 (25)	2 (15)	1 (9)	5 (8)	10 (11)
MRA time of flight	0 (0)	0 (0)	0 (0)	0 (0)	0 (0)
MRI vessel wall imaging	0 (0)	0 (0)	0 (0)	3 (5)	3 (3)
Ultrasound Doppler	0 (0)	0 (0)	0 (0)	3 (5)	3 (3)
Other (please specify)^a^	3 (38)	0 (0)	0 (0)	1 (2)	4 (4)
6. What is your preferred treatment of a carotid web in asymptomatic patients?					
Don’t know	4 (67)	2 (18)	3 (30)	5 (11)	14 (19)
Nothing	0 (0)	4 (36)	1 (10)	12 (26)	17 (23)
Follow-up imaging only	0 (0)	0 (0)	1 (10)	1 (2)	2 (3)
Aspirin only	1 (17)	3 (27)	4 (40)	21 (45)	29 (39)
Plavix (clopidogrel) only	0 (0)	0 (0)	0 (0)	0 (0)	0 (0)
Dual antiplatelet (aspirin/Plavix)	1 (17)	2 (18)	1 (10)	7 (15)	11 (15)
Coumadin (warfarin)	0 (0)	0 (0)	0 (0)	0 (0)	0 (0)
NOAC/DOAC	0 (0)	0 (0)	0 (0)	0 (0)	0 (0)
Stent	0 (0)	0 (0)	0 (0)	0 (0)	0 (0)
Endarterectomy	0 (0)	0 (0)	0 (0)	1 (2)	1 (1)
7. What is your preferred treatment of a carotid web in the setting of acute (ischemic) stroke?					
Don’t know	4 (67)	1 (9)	3 (30)	4 (9)	12 (16)
Nothing	0 (0)	0 (0)	0 (0)	5 (11)	5 (7)
Follow-up imaging only	0 (0)	0 (0)	0 (0)	0 (0)	0 (0)
Aspirin only	0 (0)	5 (45)	3 (30)	11 (23)	19 (26)
Plavix (clopidogrel) only	0 (0)	0 (0)	0 (0)	0 (0)	0 (0)
Dual antiplatelet (aspirin/Plavix)	1 (17)	1 (9)	3 (30)	14 (30)	19 (26)
Coumadin (warfarin)	0 (0)	1 (9)	0 (0)	0 (0)	1 (1)
NOAC/DOAC	0 (0)	1 (9)	0 (0)	2 (4)	3 (4)
Stent	0 (0)	0 (0)	1 (10)	9 (19)	10 (14)
Endarterectomy	1 (17)	2 (18)	0 (0)	2 (4)	5 (7)
8. What is your preferred treatment of a carotid web in the setting of multiple recurrent (ischemic) strokes?					
Don’t know	3 (50)	3 (27)	4 (40)	4 (9)	14 (19)
Nothing	0 (0)	0 (0)	0 (0)	2 (4)	2 (3)
Follow-up imaging only	0 (0)	0 (0)	0 (0)	0 (0)	0 (0)
Aspirin only	0 (0)	0 (0)	0 (0)	2 (4)	2 (3)
Plavix (clopidogrel) only	0 (0)	0 (0)	0 (0)	0 (0)	0 (0)
Dual antiplatelet (aspirin/Plavix)	0 (0)	0 (0)	0 (0)	5 (11)	5 (7)
Coumadin (warfarin)	0 (0)	1 (9)	0 (0)	0 (0)	1 (1)
NOAC/DOAC	1 (17)	0 (0)	0 (0)	1 (2)	2 (3)
Stent	1 (17)	3 (27)	3 (30)	22 (47)	29 (39)
Endarterectomy	1 (17)	4 (36)	3 (30)	11 (23)	19 (26)

Notes: All data are reported as n (%). Questions 1, 4, and 5 allowed for multiple responses (“select all that apply”), reflected in the percentages in the All category.

^a^Other responses included “Have not personally encountered,” “Never heard of carotid web,” and “I do not ever decide on how to work up carotid webs.”

CTA, computed tomography angiography; DSA, digital subtraction angiography; MRA, magnetic resonance angiography; MRI, magnetic resonance imaging; NOAC/DOAC, novel oral anticoagulant/direct-acting anticoagulant.

### Preferred Imaging Modalities

**Figure 1.**
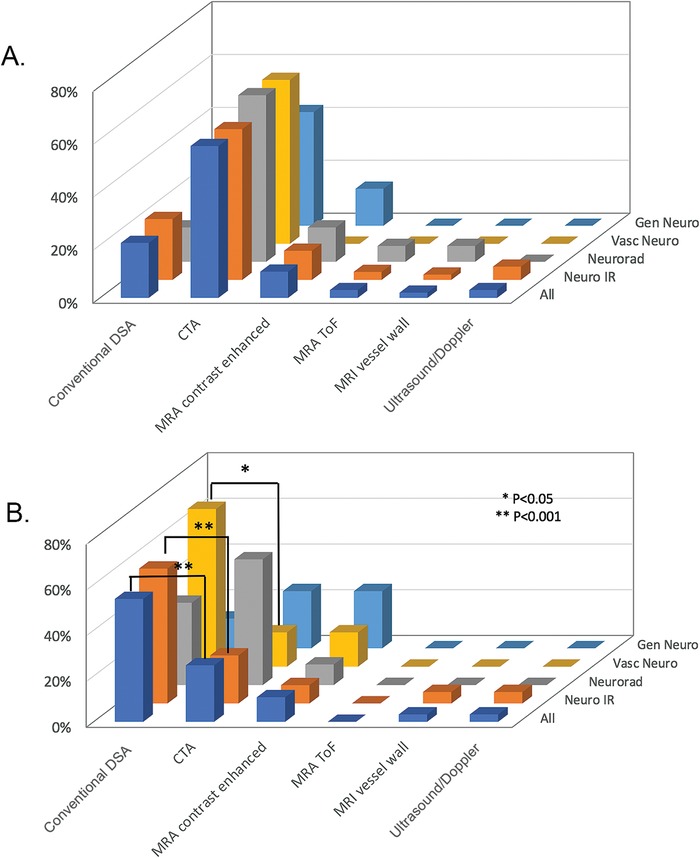
**Preferred imaging modalities (multiple responses were allowed). A. Imaging modalities used to put carotid web in the differential diagnosis (even if further imaging was sought). B. Imaging modalities used to confirm the diagnosis of carotid web.** CTA, computed tomography angiography; DSA, digital subtraction angiography; Gen Neuro, general neurologist; MRI, magnetic resonance imaging; MRA, magnetic resonance angiography; Neuro IR, neurointerventionalist; Neurorad, neuroradiologist; TOF, time of flight; Vasc Neuro, vascular neurologist.

The imaging modality that most commonly put carotid web in the differential diagnosis was computed tomography angiography (57% of responses) (Figure 1A), and conventional digitally subtracted angiogram (DSA) was the second most common (21% of responses) (Figure 1B). The preferred modality to confirm a carotid web was DSA with 54% of responses.

### Management of Carotid Webs

**Figure 2.**
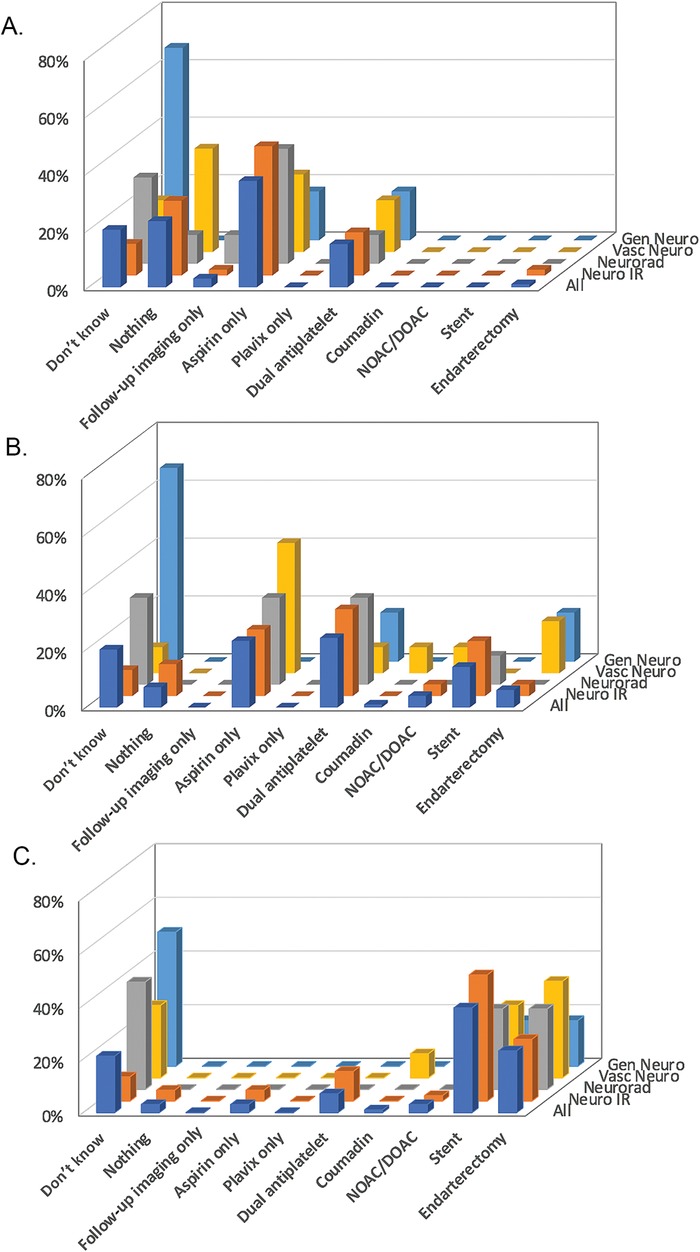
**Preferred management of carotid webs (one answer choice only; multiple selections were not allowed). In the setting of (A) asymptomatic carotid web, (B) acute stroke, and (C) multiple or recurrent strokes.** Gen Neuro, general neurologist; Neuro IR, neurointerventionalist; Neurorad, neuroradiologist; NOAC/DOAC, novel oral anticoagulant/direct-acting anticoagulant; Vasc Neuro, vascular neurologist.

For management of an asymptomatic carotid web, aspirin only was the most common response (39%), followed by nothing (23%), and don’t know (19%), with only a single vote for intervention via stent or endarterectomy (Figure 2A). For management of an acute stroke, dual antiplatelet therapy and aspirin-only treatment were tied at 26% of total responses each (Figure 2B). Management preferences for multiple or recurrent strokes overwhelmingly favored intervention, with 65% of total responses favoring stent or endarterectomy (Figure 2C), but no statistical difference favored either specific therapy.

## DISCUSSION

The fact that 57% of respondents preferred CTA for diagnosis of carotid webs suggests that the lesion is better characterized with greater sensitivity on CTA than with other noninvasive imaging techniques such as carotid ultrasound or magnetic resonance angiography (MRA). The carotid bifurcation is a common location for dephasing artifact on time-of-flight MRA, likely limiting its utility in diagnosing lesions in this specific location. Despite the widespread prevalence of carotid ultrasound use in stroke management, ultrasound was not a popular choice among our respondents. Possible reasons may include the user-dependent nature of the modality and the lack of flow-limiting stenosis on Doppler measurements.

Catheter-based angiograms were most commonly preferred for confirmation by the specialists who perform these procedures (neurointerventionalists and vascular neurologists), while noninterventional subspecialists preferred CTA for confirmatory imaging. The preference among neuroradiologists for CTA to confirm a carotid web was ≥30% higher than in every other subspecialist group. Neuroradiologists possibly have the most CTA experience among the subspecialties and therefore the most confidence in their ability to use this imaging modality to confirm a carotid web.

Identification of carotid webs as an incidental finding in asymptomatic patients is of uncertain clinical significance. Management choices were conservative across response groups. Many respondents opted for no treatment (responses for “nothing” and “follow-up imaging only” totaled 26%), and only 1 respondent indicated surgical or endovascular intervention. Medical management was a common choice among all subspecialists, either single (aspirin only) or dual antiplatelet therapy. This response mirrors the recommendation for atherosclerotic disease management.^15^ While no evidence currently exists for the role of single or dual antiplatelet therapy with carotid webs, many subspecialists may have chosen to mirror a similar disease process treatment protocol.

Preferred management in the setting of acute ischemic stroke produced the most varied responses. Single (aspirin only) and dual antiplatelet therapy were the most common responses, accounting for 26% of respondents each). Anticoagulation (warfarin and novel oral anticoagulant [NOAC]/direct-acting anticoagulant [DOAC]) received more responses in the setting of acute stroke compared to management of asymptomatic patients, while the no-treatment (“nothing”) responses decreased sharply compared to the asymptomatic patients. Responses for invasive management options increased for this population (stent and endarterectomy combined at 21% of respondents) compared to asymptomatic management. The reason why neurointerventionlists preferred stenting whereas vascular neurologists preferred endarterectomy is unclear.

Intervention was heavily favored in the setting of multiple recurrent strokes (stent and endarterectomy combined at 65% of respondents), with a split between endovascular stenting and surgical endarterectomy. Neurointerventionalists prefer stenting. Vascular neurologists prefer endarterectomy. The reason for this preference is unclear but may relate to referral patterns and specialty-specific familiarity with these procedures.

This study has limitations. The total number of respondents could be higher. Posting the survey link on a society board led to the disproportionate representation of neurointerventionalists. This disproportionate representation may have also swayed the respondents toward those in large hospital-based practices and academic centers. A more heterogeneous respondent pool would likely offer a more accurate reflection on the current practice across the clinical spectrum. Another drawback is the self-selecting nature of a response survey. The general familiarity with the disease entity may be overstated in our responses, as physicians with little to no knowledge may be hesitant to participate.

## CONCLUSION

Familiarity with carotid webs varies among specialties with increasing recognition among subspecialists, particularly neuroradiologists. CTA was clearly the most common imaging modality used when first identifying carotid webs; DSA is a viable option for confirmatory purposes. Respondents preferred no treatment or medical management for asymptomatic webs. Carotid web management in the setting of acute stroke yielded the most varied responses, and respondents showed a clear preference for intervention vs medical management for patients with recurrent strokes. The type of interventional management varied between subspecialties.

Carotid webs are a potential etiology for stroke entity, particularly cryptogenic strokes in younger patients without typical risk factors. While carotid webs remain underrecognized, subspecialists are becoming increasingly aware of this disease entity. This survey indicates that management practice patterns appear to be emerging; however, further research will be essential in the development and validation of these treatment strategies.

## Notes

This article meets the Accreditation Council for Graduate Medical Education and the American Board of Medical Specialties Maintenance of Certification competencies for Patient Care and Medical Knowledge.
